# Personalized pulmonary rehabilitation program for patients with post‐acute sequelae of COVID‐19: A proof‐of‐concept retrospective study

**DOI:** 10.14814/phy2.15931

**Published:** 2024-01-31

**Authors:** Wendel Dierckx, Wilfried De Backer, Yinka De Meyer, Eline Lauwers, Erik Franck, Jan De Backer, Kris Ides

**Affiliations:** ^1^ Centre for Research and Innovation in Care, Faculty of Medicine and Health Sciences University of Antwerp Antwerp Belgium; ^2^ Multidisciplinary Medical Centre MedImprove BV Kontich Belgium; ^3^ Laboratory of Experimental Medicine and Paediatrics, Faculty of Medicine and Health Sciences University of Antwerp Antwerp Belgium; ^4^ FLUIDDA NV Kontich Belgium; ^5^ Clinical Operations FLUIDDA NV Kontich Belgium; ^6^ FLUIDDA Inc. New York New York USA; ^7^ CoSys Research Lab, Faculty of Applied Engineering University of Antwerp Antwerp Belgium; ^8^ Flanders Make Strategic Research Centre Lommel Belgium; ^9^ Department of Paediatrics Antwerp University Hospital Edegem Belgium

**Keywords:** COVID‐19, functional respiratory imaging, PASC, pulmonary rehabilitation

## Abstract

Long‐COVID patients present with a decline in physical fitness. The aim of this study is to reveal the impact of pulmonary rehabilitation (PR) on physical fitness, quality of life (QoL), and parameters of quantified thorax CT. Long‐COVID patients enrolled in a 3‐month PR program were retrospectively studied. PR included endurance and resistance training three times a week. Assessments pre‐ and post‐rehabilitation included quantified chest CT, pulmonary function tests (PFT), six‐minute walk test (6MWT), cardiopulmonary exercise test, and questionnaires: Hospital Anxiety and Depression Scale, post‐COVID‐19 Functional Status scale, Borg score, and EuroQol. Seventeen subjects (5M/12F), mean age 42 ± 13 years, were included. PR improved all questionnaires' results significantly. Only significant difference in PFT parameters was correlation between baseline total lung capacity (TLC) and difference in TLC pre‐ and post‐rehabilitation (*p* = 0.002). 6MWT increased from 329 to 365 m (*p* < 0.001), VO2max changed from 21 to 24 mL/kg/min (*p* = 0.007), peak load increased from 116 to 141 Watt (*p* < 0.001). Blood volume in small pulmonary vessels of 1.25 to 5 mm^2^ in cross‐sectional area (BV5%) was higher than observed in patients with acute COVID‐19 infection. After rehabilitation, BV5% decreased from 65% to 62% (*p* = 0.020). These changes correlated directly with changes in TLC (*p* = 0.039). Quantified CT airway volume increased after rehabilitation (*p* = 0.013). After rehabilitation, TLC tended to normalize due to (re)opening of small airways, with decline in air trapping and recruitment of alveoli. Furthermore, this study revealed that pulmonary rehabilitation can improve QoL and physical fitness in long‐COVID patients.

## 1 TAKE HOME MESSAGE

After rehabilitation, significant (re)opening of the small airways was observed, resulting in decline in air trapping and recruitment of alveoli. Rehabilitation had a significant impact on quality of life and cardiorespiratory fitness of patients with PASC.

## 2 BACKGROUND

In 2019, the coronavirus SARS‐CoV‐2 was at the root of a pandemic (Ciotti et al., 2020). Common signs and symptoms included fever, cough, and tiredness (Ochani et al., 2021).

Many people continue to experience symptoms beyond the infection's initial phase. Long‐COVID or these post‐acute sequelae of COVID‐19 (PASC), affect COVID‐19 survivors at all levels of disease severity, including younger adults, children, and individuals who are not hospitalized (Yong, 2021).

According to current definitions, PASC is a condition marked by symptoms that continue more than 4 weeks after the start of SARS‐CoV‐2 infection symptoms (Fernández‐de‐las‐Peñas, 2022). There are two types of it: “subacute symptoms” are the symptoms and indications that continue 4 to 12 weeks after the acute infection and “chronic PASC” refers to the lingering symptoms that continue 12 weeks after the original onset. There is a great deal of variability in this chronic illness (Datta et al., 2020), with over 100 long‐term COVID symptoms compiled in systematic reviews. The most prevalent ones are dyspnoea and exhaustion, but reports of cognitive impairment (memory loss, brain fog), chest pain, and joint pain are also common. (Daines et al., 2022; Hayes et al., 2021; Lopez‐Leon et al., 2021).

Reduced physical fitness and a decline in quality of life (QoL) are the results of these and other chronic ailments (Tabacof et al., 2022).

The prevalence of PASC has been estimated to be 43% of patients who have experienced an acute infection. With this, there are greater risks for patients who were hospitalized during acute COVID‐19 infection compared with nonhospitalized patients (49% vs 37%) (Chen et al., 2022).

No guidelines are currently available for the treatment of PASC (Kim et al., 2022; Yelin et al., 2022). Many papers here are finding evidence of positive results to provide pulmonary rehabilitation (PR) to patients with PASC (Veronese et al., 2022). However, systematic reviews report poor quality papers with little or missing data (Fugazzaro et al., 2022; Piva et al., 2023; Soril et al., 2022). Most evidence is focused on patients hospitalized during their acute infection, and little is known about the impact of PR in nonhospitalized patients (Soril et al., 2022). These patients are the focus of this paper. The primary aim is to evaluate the impact of PR on physical fitness and quality of life through a comprehensive assessment before and after rehabilitation. The secondary aim was to evaluate the impact of PR on parameters obtained with quantified thorax CT.

## 3 MATERIALS AND METHODS

### 3.1 Study design

A retrospective study was conducted in the multidisciplinary medical practice MedImprove in Kontich (Flanders, Belgium). All subjects in this practice signed an informed consent approving that their medical data can be pseudonymized and utilized for research purposes. This monocentre study was reviewed and ethically approved by the ethics committee of the University Hospital of Antwerp on December 12th, 2022 (Project ID 5050).

Previous research revealed that patients who do not receive therapy show no improvement (Torres & Gradidge, 2023). Therefore, no control group was included in this study. Furthermore, failure to treat patients who seek help after a long period of post‐COVID symptoms is unethical.

A discrete sample size will be included since all patients were extensively evaluated using classical outcome measures as well as outcomes from an imaging technique that is more sensitive than the endpoints of pulmonary function tests (De Backer et al., 2012).

### 3.2 Participants

#### 3.2.1 Inclusion and exclusion criteria

Between August 2021 and October 2022, data from MedImprove patients who had respiratory symptoms that persisted for at least 4 weeks following their initial COVID‐19 infection were gathered. All consecutive male and female patients >18 years of age who were willing to participate in a 3‐month pulmonary rehabilitation program and provided signed informed consent were enrolled. Individuals who had prior heart or lung conditions, myalgic encephalomyelitis/chronic fatigue syndrome (ME/CFS), or other comorbidities that could have interfered with long‐term COVID‐19 symptoms were excluded from the study. In addition, subjects who did not complete the pulmonary rehabilitation program until the end were excluded from the study.

### 3.3 Measurements and endpoints

In this retrospective study, all patients received a standardized battery at intake.

Demographic data were collected, including age, sex, height, and weight. Medical history was recorded, including vaccination status, onset of acute COVID‐19 infection (positive PCR) and treatment.

After general clinical examination, assessment of current symptoms and concomitant medication, the baseline measurements were executed.

All patients underwent a pulmonary function test (PFT) (Vyntus Body, Vyaire Medical).

Non‐contrast, chest CT was acquired at total lung capacity (TLC) and functional residual capacity (FRC) and post processed by FLUIDDA (Detailed description in Data S1).

If the clinical condition allowed (e.g., no desaturation) and no major deviations on FRI were observed (e.g., serious declined BV5% as seen in acute COVID infection (Lins et al., 2020)), patients performed a cardio pulmonary exercise test. The cardiorespiratory fitness (VO2 peak in mL/kg/min) was measured by a maximal incremental cardiopulmonary exercise test (CPX) using a Vyntus CPX device (Vyaire Medical) and an ergo cycle (GE Vivid S60 N NOR v204) with monitoring of ECG, saturation and blood pressure (Cardiosoft GE).

Other physical tests performed were the 6‐minute walk test (6MWT), handgrip force (HGF) and quadriceps force (QF), and Borg Dyspnoea Scale (Borg). Patients were asked to complete the following questionnaires: the EuroQol Questionnaire (EQ), Hospital Anxiety and Depression Scale (HADS), and post‐COVID‐19 Functional Status scale (PCFS) (Klok et al., 2020). The latter is a global instrument that correlates with the quality of life, dyspnoea and mental health and is also an appropriate tool for screening patients requiring careful follow‐up after COVID‐19 infection (Benkalfate et al., 2022). It categorizes patients into 4 groups from neglectable (Grade 1) to severe (Grade 4) functional limitations. The purpose of the questionnaires was to evaluate the impact of their disease and rehabilitation program on their quality of life (QoL).

All tests were performed before and after a personalized 3‐month pulmonary rehabilitation program.

### 3.4 Interventional METHODS

A personalized pulmonary rehabilitation program was composed based on the measurements described in the previous section. The program consisted of cycling, treadmill walking, arm exercises, strength training, and inspiratory muscle training. The setting for cycling was set at 50% of the load reached at the anaerobic threshold during the CPX. The program started with intervals of 2 min with 30 s of rest in between. The walking speed on the treadmill was based on the distance of the 6MWT. Initially, the speed was set at 60%. Arm ergometric exercise was started in intervals of 1.30 min and 30 s pauses. All cardio exercises were initially set at 10 min for each exercise.

One repetition max (1RM) was determined to set the weight for muscle training at 60% of the 1RM. A set of 8 repetitions was used to start.

All exercises were programmed in Technogym software and transferred to a personal key.

Inspiratory muscles were trained with powerbreath KH2 and Breathlink software (powerbreath Ltd. GB). An MIP test (Maximal Inspiratory Pressure) was performed, and training was initially set on 60% of the MIP and increased gradually over each week. Patients trained 2 or 3 times a week in our centre. If the training was set at 2 times, one extra session with home exercises was provided. This session consisted of walking, cycling and strength training. Patients were monitored by using a polar app and chest strap connected to Mywellness software (Technogym). Heartrate was monitored during both home and in‐praxistraining using an H10 polar heartrate belt. For safety reasons and follow‐up, saturation, blood pressure, and Borg score for dyspnoea and fatigue were reported before and after each training at the centre. Physiotherapist were in close contact with the patient during every visit to pick up symptoms of postexertional malaise. Together with the pulmonologist, the results were weekly discussed with the patient. Progression was set at a 10% to 20% increase in load over 1 to 2 weeks, depending on the patient's Borg score for dyspnoea and fatigue reported after training for each exercise.

### 3.5 Statistics

SPSS Statistics 28.0.1.1 (IBM) was used for analysis. All analyses were evaluated using a significance cutoff of *p* < 0.05.

Demographic data were examined descriptively to gain an understanding of the participating population. Discontinuous (nonparametric) variables were described using frequency distributions. Continuous (parametric) variables were described using the mean and standard deviation.

Given the small sample size, nonparametric tests were used to find correlations (Spearman) or differences before and after rehabilitation (Wilcoxon signed‐rank test).

## 4 RESULTS

### 4.1 Basic characteristics

Between August 2021 and October 2022, a total of 25 subjects presented at the clinical practice of MedImprove with persisting respiratory symptoms at least 4 weeks after the initial COVID‐19 infection.

At the moment of acute COVID‐19 infection, all patients were vaccinated for COVID‐19, following the vaccination scheme provided by the Belgian government. None of the patients had any respiratory disease or issues (ME/CFS) before their infection. For their acute infection, all subjects were initially treated symptomatically with paracetamol and rest, 2 subjects were administered oral prednisolone. None of the patients required hospitalization. None of the cohort was on inhaled therapy prior to their first consultation for long‐COVID.

**FIGURE 1:**
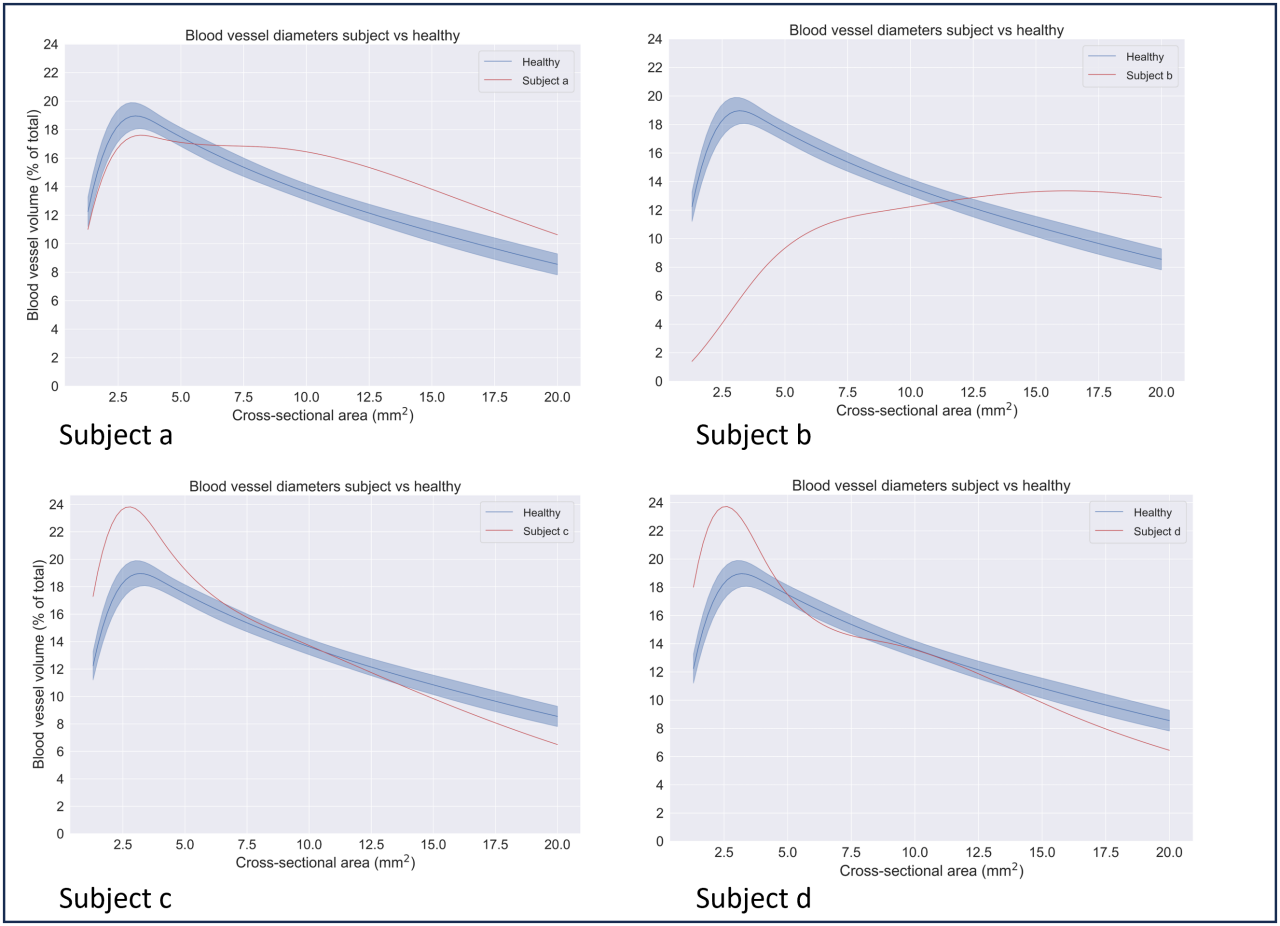
Differences in spectrum plot of BV5% at baseline. FRI revealed a declinded BV5% in subject a and b. Those subjects were excluded from the study. Subjects c and d showed an increased BV5% and were enrolled in de pulmonary rehabilitation program.

Based on the results of the baseline assessment, 2 subjects desaturated in rest and during exercise. Their CT scan revealed decreased blood vessel volumes in the small vessels, which was also observed in patients with acute COVID‐19 infection. Their spectrum plot of small blood vessels is depicted in Figure 1, together with spectrum plots of patients enrolled in the rehabilitation program.

Subject a and b were initially not enrolled in the PR program and treated with prednisolone and supplemental oxygen. They had to wait until recovery to start PR. As this was out of time for this retrospective study, they were excluded.

**FIGURE 2:**
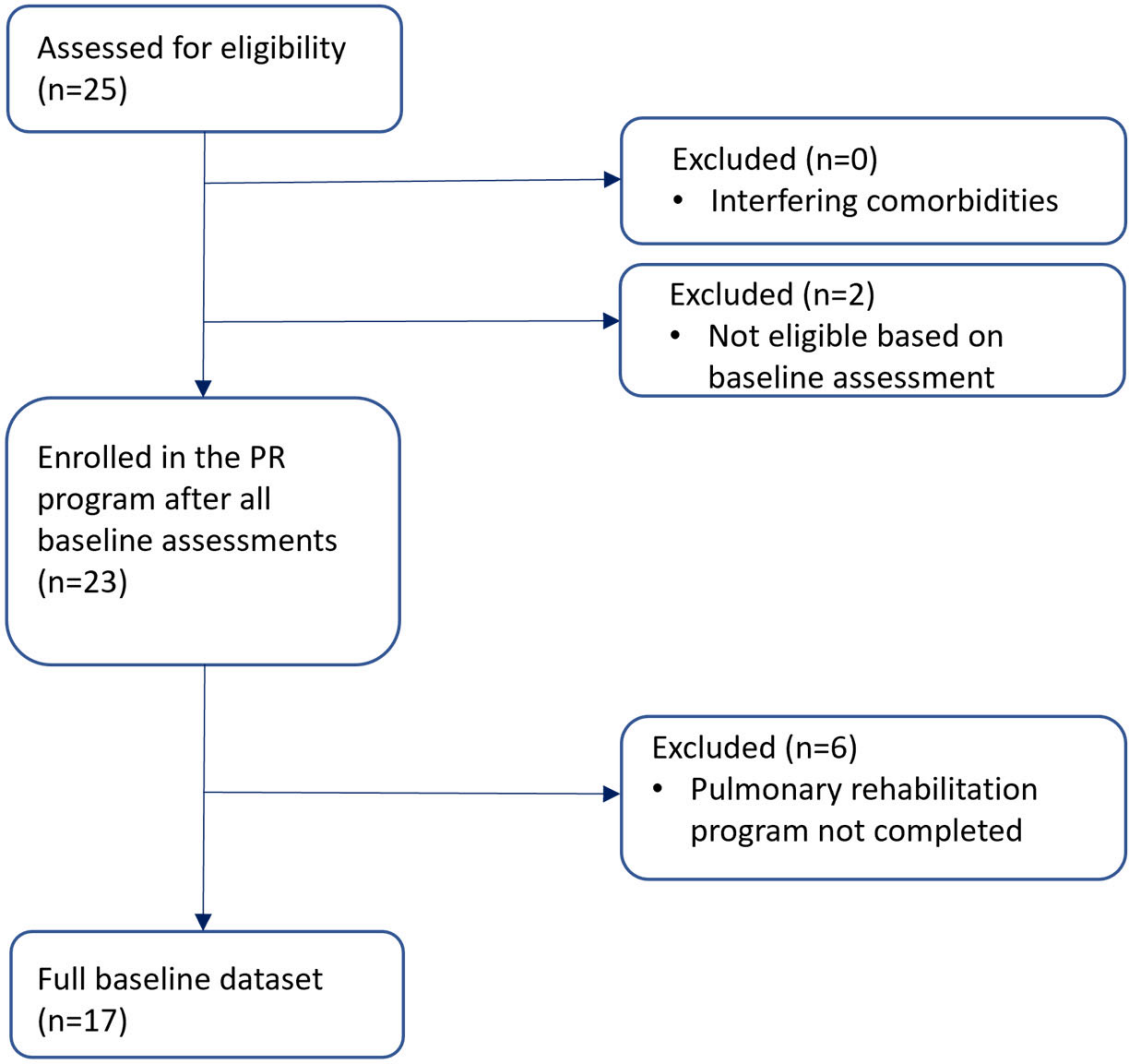
Flowchart of the enrolment.

A cohort of 23 patients was proposed to participate in the 3‐month rehabilitation program. At baseline, no subject had any complaints of postexertional malaise. Six of the subjects did not complete the program, 1 patient had a surgery on the foot, 1 patient had a fracture of the metatarsal bone, 2 patients felt better after 6 weeks and stopped rehabilitation to return to work, 2 patients stopped without giving any reason (Figure 2).

A total of 17 subjects (5 male; 12 female), with a mean age of 42 ± 13 years, were retrospectively studied. Fifteen subjects had a positive polymerase chain reaction (PCR), and two subjects tested positive on a self‐test. Five of these 17 patients were in the phase of subacute PASC, and 12 of them had chronic PASC. Descriptive data of the basic characteristics are given in Table 1.

**TABLE 1:** Descriptive statistics of basic characteristics.

		*N*	Mean	Median	Range	IQR
Age	Years	17	42	40	49	16
Gender	M/F/X	17	5 M	12 F	0 X	/
Height	cm	17	172	172	29	10
Weight	kg	17	78	75	73	24
Body Mass Index	kg/m^2^	17	26	24	21	10
Time positive PCR—first consultation	days	17	139	124	293	186

During the PR program, based on the pulmonary function test, eight of the 17 patients were treated with inhalation therapy (a combination of ultrafine long‐acting β2 mimetic (LABA) and inhaled corticosteroids (ICS)).

### 4.2 Questionnaires

At baseline, the questionnaires revealed declined mental health, and all subjects reported functional limitations. After the PR, all completed questionnaires evolved significantly to a more positive status of physical and mental health. Furthermore, the participants showed less dyspnoea (*p* = 0.005). Quality of life, measured by EuroQol questionnaires, improved (EQ index *p* = 0.003); EQ VAS (*p* = 0.004). Their mental health improved as they perceived less anxiety (*p* = 0.014) and less depression (*p* = 0.009). Their functional status measured by PCFS increased significantly (*p* < 0.001). Details presented in Table 2.

**TABLE 2:** Results questionnaires.

		*N*	Pre PR^a^	Post PR^a^	*p* Value^b^
Borg Dyspnea Scale	%	15	2 (2)	0.5 (1.5)	**0.005
EuroQol Questionnaire Index Value	%	13	0.591 (0.364)	0.833 (0.340)	**0.003
EuroQol Questionnaire Visual Analog Scale	%	13	60 (28)	70 (33)	**0.004
Hospital Anxiety and Depression Scale_Anxiety	%	12	6 (9)	3.5 (8)	*0.014
Hospital Anxiety and Depression Scale_Depression	%	13	6 (5)	1 (6)	**0.009
Post COVID Functional Status Scale	%	17	3 (1)	2 (2)	*** < 0.001

^a^ Data are presented as median (Inter Quartile Range).

^b^ Correlation is significant with *p < 0.05, **p < 0.01, and ***p < 0.001.

### 4.3 Pulmonary function test

No significant differences were found in pulmonary function test results before and after completing the rehabilitation program when comparing only the mean values (Table 3).

**TABLE 3:** Results pulmonary function test.

		*N*	Pre PR^a^	Post PR^a^	*p* Value^b^
Forced expiratory volume in 1 s (FEV1)	%	17	93 (15)	97 (13)	0.460
Forced vital capacity (FVC)	%	17	96 (14)	97 (13)	0.897
Tiffeneau index (FEV1/FVC)	%	17	78 (8)	80 (6)	0.653
Residual volume (RV)	%	17	100 (21)	103 (19)	0.463
Total Lung capacity (TLC)	%	17	96 (13)	100 (9)	0.054
Specific airway resistance (sRaw)	%	17	121 (25)	128 (26)	0.093
DLCO	%	17	95 (13)	95 (9)	0.868
Diffusing capacity (DLCO/VA)	%	17	102 (19)	95 (23)	0.055

^a^ Data are presented as the mean ± SD.

^b^ Correlation is significant with *p < 0.05, **p < 0.01, and ***p < 0.001.

**FIGURE 3:**
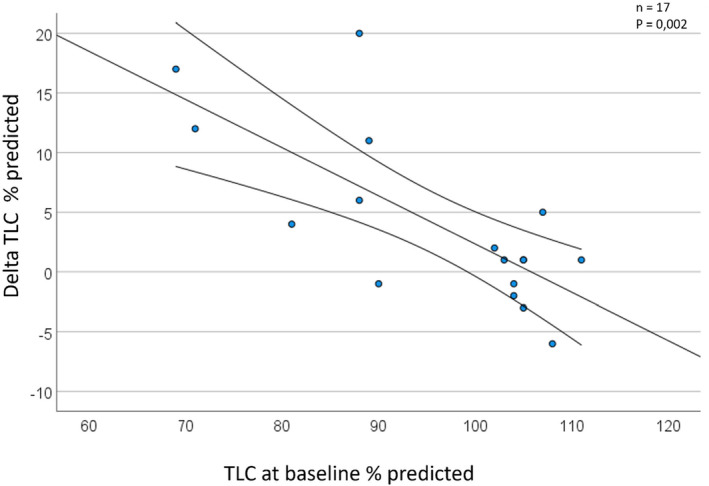
Correlation between baseline TLC and changes in TLC.

Nevertheless, a significant correlation was found between the baseline total lung capacity (TLC) and the difference in TLC before and after rehabilitation (*p* = 0.002) (Figure 3). At baseline, there was a large range in TLC percent predicted (69%–111%; range 42%). After rehabilitation, the TLC tended to decrease in patients who presented with hyperinflation and to increase in those with restrictive defects.

### 4.4 Exercise capacity

Overall exercise capacity improved significantly, as shown in Table 4. After rehabilitation, the 6‐minute walking distance increased from 497 m to 577 m (*p* < 0.001). The VO2max increased from 21 mL/kg/min to 24 mL/kg/min (*p* = 0.007), and the peak load increased from 116 Watt to 141 Watt (*p* < 0.001).

**TABLE 4:** Results exercise capacity.

			*N*	Pre PR^a^	Post PR^a^	*p* Value^b^
Handgripforce dominant side	mean (SD)	kg	17	32 (10)	34 (10)	*0.035
Handgripforce not dominant side		kg	17	28 (10)	32 (9)	*** < 0.001
Quadriceps force dominant side		kg	17	329 (125)	365 (108)	0.076
6 Minute Walk Distance (6MWD)		m	16	497 (190)	577 (151)	*** < 0.001
VO2 max		mL/kg/min	17	21 (8)	24 (8)	**0.007
O2 Pulse at peak load		mL	17	11 (2)	12 (3)	0.088
EqO2 at peak load			17	30 (5)	31 (7)	0.210
EqCO2 at peak load			17	28 (4)	29 (6)	0.162
CPX peak load		Watt	17	116 (52)	141 (56)	*** < 0.001

^a^ Data are presented as the mean ± SD.

^b^ Correlation is significant with *p < 0.05, **p < 0.01, and ***p < 0.001.

### 4.5 Chest CT

All scans were evaluated by trained radiologists. Visually, all subjects had normal CT thorax scans. There were no signs of ongoing parenchymal infiltrates or fibrotic tissue.

Changes in quantified CT parameters are presented in Table 5. The blood volume in the small pulmonary vessels of 1.25 to 5 mm^2^ in cross‐sectional area (BV5%) is higher than that observed in patients with acute COVID‐19 infection. After rehabilitation, the BV5% decreased from 648% to 624% (*p* = 0.020). These changes are directly correlated with changes in TLC (*p* = 0.039). An example of changes in the spectrum of small blood vessels before and after rehabilitation is displayed in the Data S1.

**TABLE 5:** Results quantified CT parameters.

		*N*	Pre PR^a^	Post PR^a^	*p* Value^b^
BV 5	%	16	64.8 (4.3)	62.4 (3.4)	*0.020
BV 5–10	%	16	17.8 (1.9)	18.6 (1.6)	0.121
BV 10	%	16	17.4 (2.9)	19.0 (3.2)	**0.006
Total lobe volume	L	16	5.1 (1.7)	5.3 (1.5)	*** < 0.001
Total blood vessel volume	mL	16	146.3 (25.7)	148.2 (35.6)	0.501
Total air trapping	%	14	18.6 (20.6)	16.4 (18.4)	0.925
Total airway volume	mL	16	56.3 (20.6)	60.2 (21.7)	*0.013

Abbreviations: BV 10, vessels bigger than 10 mm^2^ in cross‐sectional area; BV 5, vessels bigger than 1.25 mm^2^ and less than 5 mm^2^ in cross‐sectional area; BV 5–10, vessels bigger than 5 mm^2^ and less than 10 mm^2^ in cross‐sectional area.

^a^ Data are presented as the mean ± SD.

^b^ Correlation is significant with *p < 0.05; **p < 0.01; and ***p < 0.001.

The baseline TLC correlated significantly with air trapping measured by quantified CT (*p* = 0.048). There was no significant difference in air trapping before and after rehabilitation (*p* = 0.925). The quantified CT revealed a significant improvement in the airway volume after rehabilitation (*p* = 0.013).

**FIGURE 4:**
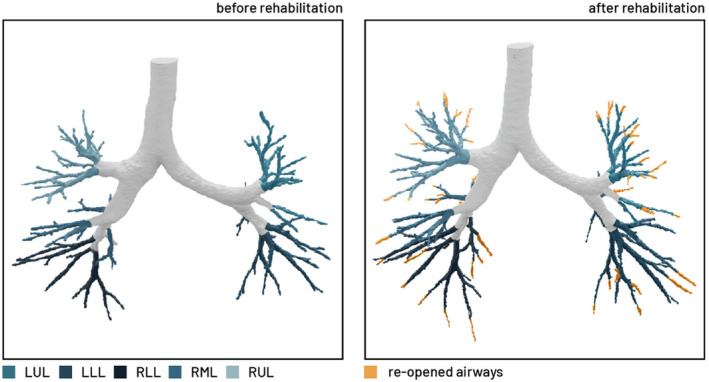
Evolution of airway volumes. Evolution in spectrum of the small blood vessels.

In Figure 4, a typical case is depicted. The increase in airway volume was evaluated in the total sample size, and no difference was found between patients with or without inhalation therapy (*p* = 0.462). The results of this study were discussed with each patient included in this study.

## 5 DISCUSSION

This retrospective study investigated the impact of a personalized PR program on physical fitness and quality of life through a comprehensive assessment before and after rehabilitation in patients with PASC. Furthermore, the impact of the PR on parameters obtained with quantified CT thorax was studied. The different endpoints assessed by PFT, CPX and quantified CT are correlated to each other, painting a picture of the physiological changes that rehabilitation can bring about.

This study revealed a general improvement in cardiorespiratory fitness measured by the CPX and 6MWT. The outcomes of the questionnaires reflected enhanced quality of life. The blood vessel volume in small blood vessels (BV5) was higher than that during acute COVID infection and normalized after rehabilitation. The increased BV5 reflects a blood volume shift from smaller vessels than BV5 to the BV5 vessels. Those smaller vessels are beyond the resolution of the scan but correspond with alveolar vessels in which gas exchange takes place. So the higher BV5 in these patients reflects suboptimal gas exchange. The changes in BV5 correlated with changes in TLC. The wide range in baseline TLC decreased due to a significant gain in imaged airway volume as given by quantified CT of the thorax.

Of the 25 patients screened for eligibility, two subjects showed a decreased BV5% on the quantified CT scan, whereas the other patients demonstrated an increase. A similar decline in BV5% was also observed in patients with acute COVID‐19 infection (Lins et al., 2020) A reduced BV5% correlates with the need for supplemental oxygen (Dierckx et al., 2022). It probably reflects a much more pronounced occlusion in smaller vessels including the alveolar vessels and the BV5 themselves. These two patients required therapy with oral prednisolone and supplemental oxygen because they demonstrated low saturation during exercise. Their condition did not allow them to safely start the pulmonary rehabilitation program. This screening is important to prevent postexertional malaise (Twomey et al., 2022). Out of the 23 subjects enrolled in the pulmonary rehabilitation program, 17 patients completed the 3‐month program and were included in this study. Most of this cohort was female (12 out of 17), reflecting the findings that females have a higher prevalence of PASC than males (49% vs 37%) (Chen et al., 2022).

Unlike pulmonary rehabilitation for COPD patients, this program started at a low training load with a gentle increase in resistance according to the patient's capabilities. We assumed that the decrease in small alveolar blood vessels (<2 mm^2^) could have a large impact on the intensity of the exercise. Hence, we started with lower loads to recruit these small vessels and to minimize training in anaerobic conditions.

The significant improvement of all questionnaires is a confirmation that pulmonary rehabilitation had a positive effect on their mental health and patient perception of functional limitations. The impact of rehabilitation on QoL is conflicting. In many studies, PR was provided as telerehabilitation. A subgroup analysis revealed that face‐to‐face PR was effective in improving QoL (Ahmed et al., 2022).

In addition to the self‐reported outcomes, the objective measurements of the exercise capacity tests revealed the same improvement. At baseline, decreased exercise tolerance was observed. Invasive CPX offers objective evidence arguing against simple deconditioning as an explanation for these symptoms (Joseph et al., 2023). The exact underlying pathophysiology is rather obscure (Munipalli et al., 2022). The improvement in VO2 max is clinically very significant, which is meaningful since it has been demonstrated that a decreased VO2 max is related to increased symptoms of PASC (Aparisi et al., 2021). Furthermore, handgrip force, six‐minute walking distance, and peak workload increased significantly. These results are comparable with findings in other studies where the included population was mainly hospitalized during acute COVID infection (Compagno et al., 2022).

There were no significant changes found in pulmonary function tests. This contrasted with earlier studies where a significant improvement in FEV1% predicted was found (Mińko et al., 2023; Nopp et al., 2022). The mean FEV1% predicted of the population at baseline was lower in these studies than in the population studied in this paper (82% and 86% vs 96%).

The most marked change in the quantified CT parameters was the substantial difference in imaged airway volume before and after rehabilitation. A significant correlation was found between the baseline total lung capacity (TLC) and the difference in TLC before and after rehabilitation (measured by body plethysmography). At baseline, a wide variation in TLC was observed (Dierckx et al., 2023). The TLC tended to decrease in patients who presented with hyperinflation. Small airway disease and air trapping were also reported in other studies where patients with PASC were evaluated with quantified CT (Cho et al., 2022). The air trapping resulting in hyperinflation could be resolved by reopening of the airways. On the other hand, in patients with restrictive defects and low TLC, the increase in airway volume resulted in recruitment of alveoli. We hypothesize that this is mainly due to rehabilitation, as no difference was observed in the increase in airway volume between patients with or without inhalation treatment.

In most patients, total lung capacity increased after the rehabilitaion. This was not reflected by the TLC measured by the pulmonary function test; however, the total lobe volume measured by quantified CT increased significantly after rehabilitation. Quantified CT was more sensitive to detect this signal.

Patients with acute COVID infection demonstrated decreased volumes in BV5 as a result of increased pulmonary vascular resistance, occlusion due to microthrombi or tissue damage. This resulted in redistribution of the blood from smaller to larger vessels. (Lins et al., 2020) In patients with PASC, the BV5 at baseline was rather elevated. The changes in BV5 before and after rehabilitation correlated with the changes in TLC. We hypothesize that compression of very small alveolar blood vessels (<1.25 mm^2^) in hyperinflated lung zones shifts the blood to larger vessels with a “peak” in BV5. By resolving the hyperinflation, BV5 can be normalized. BV5 in PASC patients can be normalized by treatment that (re)opened the airways.

This study has several limitations. The sample size used in this proof‐of‐concept study is discrete and needs to be expanded for further validation and to phenotype these patients in more detail. No control group was included in the study, based on previous research and for ethical reasons. Since the patients started rehabilitation only after they had not received therapy for several months, one could speak of a crossover design. The baseline findings are after a period without therapy and includes natural recovering, the post treatment assessments are after rehabilitation. Selection bias is another potential concern because all data were retrospectively collected in 1 centre. All included subjects had chosen to present to a clinic with post‐COVID symptoms, which might introduce unmeasured confounders. Most patients were suspected of being infected with the Omicron variant; however, this was not always confirmed and therefore not included in the manuscript. In this study, no blood parameters were included. Blood analysis could be used in additional research to verify the findings. The quantified CT has no reference values but can be used for interpatient comparisons.

This study focused on long‐COVID patients who were not hospitalized during their acute COVID‐19 infection. Future research will be needed to confirm these findings in a larger sample, also including patients who were hospitalized during their acute COVID‐19 infection. Furthermore, the patients previously excluded from enrolment in a personalized rehabilitation program due to unstable conditions should be evaluated again and studied in the same manner.

## 6 CONCLUSIONS

A large number of patients are confronted with the long‐term sequelae of COVID infection. Although hospitalization is reported as an important factor for post‐COVID symptoms, there is a large cohort of nonhospitalized patients who present with the long‐term effect of a COVID infection. In this study, an adapted and personalized PR program was used to tackle these effects, focusing on a number of symptoms by dealing with the patients' fatigue, exercise tolerance and postexercise malaise. A positive change in exercise tolerance, TLC, QoL and airway volume was noticed after a personalized 3‐month PR program. A thorough assessment, consisting of a combination of questionnaires, CPX and functional testing, to build and provide a patient‐tailored PR program, provided satisfaction effects in patients suffering from PASC after a COVID infection. This assessment can be used for the follow‐up and motivation of patients during their rehabilitation, as it provides the necessary feedback to patients about their progression.

## FUNDING INFORMATION

This study was executed without funding.

## DECLARATION

All authors have read and approved the manuscript.

## ETHICS STATEMENT

All subjects in this practice signed an informed consent approving that their medical data can be pseudonymized utilized for research purposes. This monocenter study was reviewed and ethically approved by the Ethical Committee of the University Hospital of Antwerp on December 12, 2022 (Project ID 5050).

## Supporting information


Data S1.
