# Supplementary material for: Personalized pulmonary rehabilitation program for patients with post‐acute sequelae of COVID‐19: A proof‐of‐concept retrospective study
Source: Physiol Rep. 2024 Jan 31;12(3):e15931. doi: 10.14814/phy2.15931 (PMC10830387; doi:10.14814/phy2.15931)
Supplement: Supplementary file 1 — Data S1. [file PHY2-12-e15931-s001.docx]

**Supplemental Digital Content**

***Functional Respiratory Imaging methodology***

Noncontrast, thin-slice, volumetric chest CT was acquired at total lung capacity (TLC) and functional residual capacity (FRC) and postprocessed by FLUIDDA.

3D reconstructions of the lungs and pulmonary vasculature were created by FLUIDDA. An automated blood vessel segmentation algorithm performed an eigenvalue analysis of the Hessian matrix to enhance and identify tubular structures by returning the probability of each voxel belonging to a tubular structure based on shape analysis ^1^. Next, Hounsfield unit (HU) thresholds are used to limit the vessels. The HU thresholds are based on the vessel size and are defined by an automated adaptive iterative threshold method. In the preprocessing, a gradient anisotropic diffusion filter is applied, and a region of interest is defined to remove some false positives. Subsequently, the smaller nonconnected parts are removed. To account for the effects of slice thickness on the results, sensitivity analysis was performed. Volumes were then computed from the cross-sectional area of each vessel. Following the convention established by Rahagi et al., the volume of blood contained in vessels below 5 mm^2^ cross-sectional area (down to a cutoff of 1.25 mm^2^) was termed “BV5”. Additionally, BV5-10 was defined as the volume of blood contained in vessels with cross-sectional areas between 5 and 10 mm^2^, and BV10 was defined as the volume contained in vessels with cross-sectional areas above 10 mm^2^. We refer collectively to these quantities as BVX. To account for variation in lung volume, we chose to normalize BVX by total pulmonary blood volume. This permits for the computation of a “BV spectrum”, a curve representing the percent of total pulmonary blood volume contained within vessels of a given calibre as a function of cross-sectional area. It had previously been observed in the analysis of scans from healthy volunteers that this yielded values with very low variance over all scales.

Functional respiratory imaging-based air trapping is defined as all intrapulmonary voxels with Hounsfield units between -1024 and -850 using expiratory scans at FRC. A binomial blur filter is applied, and a mask within these thresholds is created, intersecting with each lobe mask to make the regional air trapping masks. The 3D models are then created from these masks.

By identifying and grouping the voxels that represent the air in the lungs, the lung volume (L) could be determined from the scans.

The airways were segmented up to the point where no distinction can be made between the intraluminal and alveolar air. This is where the airway diameter is approximately 1 – 2 mm, typically around the 5th to 10th bifurcation, depending mainly on the disease state of the individual patient ^2^.

**An example of changes in the spectrum of small blood vessels before and after rehabilitation**


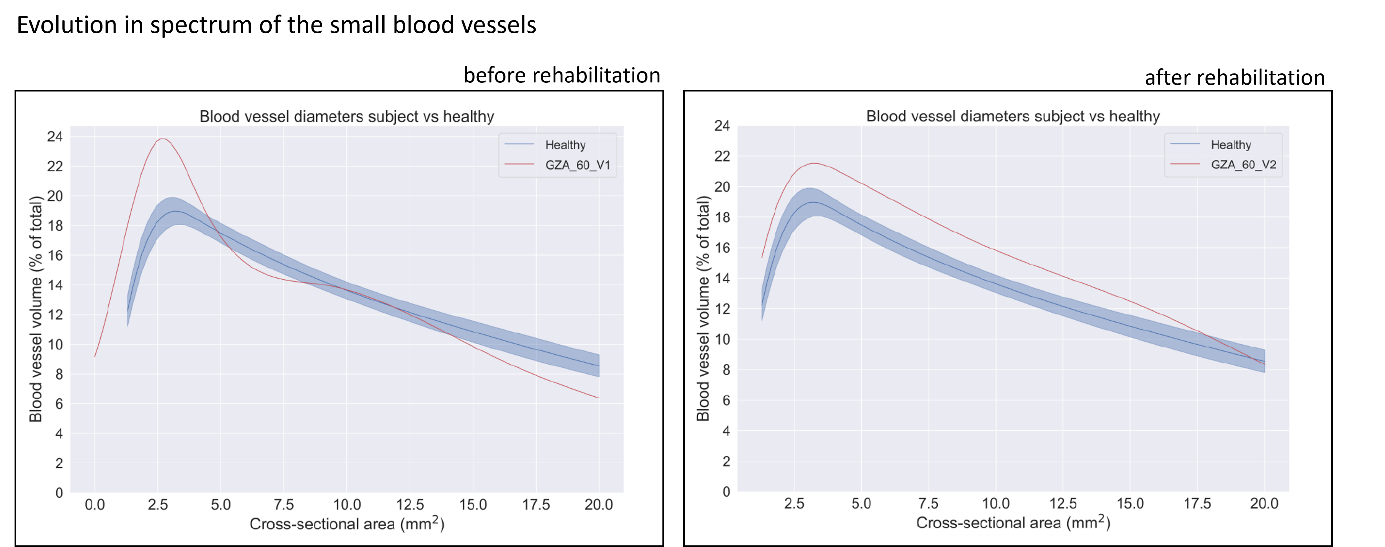


## **REFERENCE**

1. Yang J, Ma S, Sun Q, et al. Improved Hessian multiscale enhancement filter. In: *Bio-Medical Materials and Engineering*. Vol 24. IOS Press; 2014:3267-3275. doi:10.3233/BME-141149

2. Dierckx W, De Backer W, Ides K, et al. Unraveling pathophysiologic mechanisms contributing to symptoms in patients with post‐acute sequelae of COVID‐19 (PASC): A retrospective study. *Physiol Rep*. 2023;11(12). doi:10.14814/phy2.15754
